# Home Musical Activities Boost Premature Infants’ Language Development

**DOI:** 10.3390/children11050542

**Published:** 2024-05-01

**Authors:** Fabia Franco, Maria Chifa, Nina Politimou

**Affiliations:** 1 Psychology Department, Faculty of Science and Technology, Middlesex University, London NW4 4BT, UK; f.franco@mdx.ac.uk; 2 Department of Psychology and Human Development, IOE Faculty of Education and Society, University College London, London WC1H 0AA, UK

**Keywords:** language development, prematurity, musical activities, parenting

## Abstract

Infants born prematurely are considered at risk for language development delay and impairments. Using online parental reports, the present study investigated the influence of early musical experience in the home environment (Music@Home Infant Questionnaire) on language development (MacArthur–Bates Communicative Development Inventory) while controlling for general enrichment at home (Stim-Q Cognitive Home Environment Questionnaire) and perinatal post-traumatic stress disorder (Perinatal PTSD Questionnaire). Caregivers of 117 infants between 8 and 18 months of age (corrected age) without reported developmental difficulties completed an online survey. Results revealed that the musical home environment significantly predicted outcomes in reported infants’ receptive vocabulary and gestural communication, independently from infants’ corrected age and general enrichment of home activities. These findings constitute the first evidence that an enriched musical experience can enhance the development of early communication skills in a population at risk for language delays, namely infants born prematurely, opening the path for future intervention research in home and/or early childcare settings. Given that the majority of participants in this study were highly educated and from socioeconomically stable backgrounds, considerations regarding the generalizability of these results are discussed.

## 1. Introduction

The more recent available worldwide statistics [1] reported that an estimated 13.4 million babies were born preterm in 2020. This infant population often experiences language delays as documented across cultures [2,3,4,5,6,7], and risks of delays or impairments in cognitive and language development are generally more prevalent in preterm than full-term births [8,9]. The aetiology of these weaknesses is likely to be multifactorial [10], with risk factors being related to preterm birth and the iatrogenic effects of neonatal intensive care [11]. For instance, Cusson [12] investigated infants born <36-week gestation and <2 kg birth weight, assessing various infant and caregiver measures in the early months. The results showed that factors such as length of hospitalisation, birth weight, Apgar newborn scores, irritability, and state regulation when discharged from hospital—all had an influence on language outcomes. Furthermore, the last trimester of gestation is crucial for foetal brain development, with rapid development of neurons and wiring [13]. However, the interruption caused by premature birth may slow down this process. In fact, the link between the level of prematurity and language outcomes has been found to be inverse linear, in that the earlier a foetus is born, the poorer language skills s/he might have, i.e., the higher the risk for language delays [14].

A large body of research has been researching the frequency and nature of language delays in preterm children. For example, D’Odorico et al. [15] showed that Italian premature infants observed at 12, 18, and 24 months had reduced involvement in the language process and poorer receptive and productive vocabularies as measured by the MacArthur–Bates CDI (Italian adaptation) compared to their full-term peers. Using the MacArthur–Bates CDI (French adaptation) with a large sample of toddlers, Gayraud and Kern [16] showed that their preterm sample (i.e., born extremely or very preterm) produced fewer words, and their vocabulary included phonetically simpler words than their full-term peers at 24 months. This pattern also emerged from longitudinal studies. For example, Nguyen et al. [6] investigated the language acquisition trajectory in a large sample of preterm and full-term children from 2 to 13 years of age, assessing the participants at 2, 5, 7, and 13 years, using an age-suitable measure at each measurement point. Prematurely born children were shown to have poorer performance across all components of language, even at age 13. Despite the literature showing that preterm children’s language development will catch up with time [17,18], early severe delays, which are more common in the lowest gestational age group, predict a higher risk for persistent language delays throughout the preschool years, and such delays may be linked further to academic struggles as well as socioemotional and behavioural issues [19].

The environment that preterm infants are exposed to after birth is different compared to full-term infants as both lack high exposure to the human voice, which has been shown to be crucial for various developmental measures [20,21,22], including language development [23] and by being immersed in an acoustically aversive soundscape while in the Neonatal Intensive Care Unit (NICU) [24]. This environment is abundant in irregular, unpredictable stimuli, making prematurely born infants prone to hyperstimulation [25]. Therefore, parents are encouraged to get familiar with babies’ cues and notice any distress that they display, thus allowing them to moderate the kinds and quantity of stimulation that they provide to their infants [26]. In the NICU, the voices, when present, are masked by electronic, non-biological sounds, which may occur 24 h a day, making it difficult to distinguish foreground from background sounds at 60 Db or to filter out and process noxious stimuli [27,28]. Voices can also positively affect cardiorespiratory stability [29] and feeding and growth factors [30], and they have been found to reduce infant pain thresholds [31]. Yet, despite being so important, voices might even be excluded by the incubator [27,28]; however, this allows the penetration of a higher-than-desirable level of mechanical noises from medical machines [32]. Parent–child interaction can also be drastically impoverished in that caregivers might not have the opportunity to notice and respond to the infant’s cues while also being impeded by the medical equipment surrounding them or the clinical state of the infant, which may prevent them from exercising their ability to provide the quality and quantity of experiences that a full-term infant might benefit from.

For parents, having a child born prematurely can cause stress and impaired role adaptation, with parents of preterm infants being at risk of developing post-traumatic stress disorder (PTSD) symptoms [33]. Specifically in association with sound, Chifa et al. [24] revealed that parents who spent time in the NICU with their infants reported PTSD symptomatology also in association with the aversive NICU soundscape and that they felt that their communication with the baby was affected by such noises. Parental mental health may impact not only parenting skills in general but also specifically on the set of communicative adaptations pertaining to the infant-directed register (ID register henceforth), thus potentially hampering infants’ language development. For instance, Lam-Cassettari and Kohlhoff [34] found that depressed mothers used ID speech less frequently compared to non-depressed mothers, and D’Odorico and Jacob [35] showed an attenuated ID register in the speech of mothers of infants identified as ‘late talkers’. Consistently, various studies demonstrated that infants who experienced less infant-directed speech input showed poorer language development by their second year of life [36,37]. Although premature infants show the same preferences as infants born at full-term for the ID register [38], they might spend the first weeks or months in the hospital. Hence, their exposure to infant-directed speech and song can be dramatically reduced. Oyetunji and Chandra [39] found that maternal postpartum stress negatively affected infants’ language development, as well as other variables such as sleep, growth, gross motor skills and feeding (see also a systematic review based on 122 studies) [40]. Thus, parental mental health is an important variable to consider when investigating environmental factors that may impact preterm children’s language development.

On the other hand, it is possible that caregivers, being aware of their preterm infant’s vulnerability, might put extra effort into providing optimal stimulation, hoping to compensate for the initial difficulties, which might lead to the infant’s higher-than-expected cognitive functioning [41]. These two alternative scenarios (impoverished vs. compensatory) may interact with the socioeconomic status (SES) and educational levels of the caregivers. For instance, Wild et al. [42] investigated the impact that SES has on language development in preterm children born <32 weeks gestation in the US and whose families had either private insurance (P-Ins) or Medicaid-type insurance (M-Ins which is a government program providing health insurance to those from disadvantaged backgrounds in the USA). At 22 months, the language scores (Bayley Scales of Infant Development III Language Composite) were lower in preterm infants from M-Ins than in P-Ins families (87.9 ± 11.3 vs. 101.9 ± 13.6). Moreover, Patra et al. [43] suggested that at the age of 20 months, maternal education was the most powerful predictor for language development and motor and cognitive skills in premature infants. Consistently, using the CDI at 2 years corrected age, Sentenac et al. [44] found that in children born <32 weeks gestation, low maternal education significantly increased the risk of expressive language delay, namely children’s incapability of combining words and forming multi-word utterances and very poor expressive vocabulary.

Parental involvement is crucial for language acquisition, especially in the first years of an infant’s life [45]. Among activities used to engage with infants and young children, musical interaction may present a particularly opportune medium for communicating with premature infants and supporting language development. Indeed, recent research has shown a positive relationship between informal musical interactions at home and early language development in typically developing populations [46,47,48,49,50]. In early infancy, such as between 4 and 6 months of age, language patterns may be perceived as melodies and reflected in infants’ initial sound utterances [51,52]. The mastering of melodic contours, durational features, and amplitude is associated with the communication of pragmatic meanings and coupled with the advancement of phonation and articulation required for successful speech production [53,54,55]. During this early phase, social interactions can increase babbling and vocalisation if delivered contingently with infant vocalisations [56]. In the second part of the first year, the ability to discriminate non-native language-specific sounds starts to decrease while perception specialises for native language-specific phonemes (perceptual reorganisation–amongst others, see Kuhl [57]). However, novel phonetic contrasts continue to be discriminated past the phonetic narrowing window when presented in a musical context [58], thus suggesting a potentially important role of musical communication in key aspects of language development.

Besides the studies suggesting that musical experience in infancy and childhood is associated with better communication and language development outcomes [59,60,61], other aspects of parent–child interaction have also been reported to positively impact language development. For instance, studies have demonstrated that the quality of shared activities between children and their caregivers and the availability of learning materials can benefit language development and school readiness by providing more learning opportunities for children [62,63,64]. Shared picture book reading can be particularly beneficial for language acquisition [65,66,67]. Although the majority of parents report that they start to read to their infants when they are approximately 6 months [68], in the case of preterm birth, there are various programs, such as the Newborn Individualized Developmental Care and Assessment Program (NIDCAP), which encourage parents to read to their infants soon after birth. Book reading facilitates infants’ exposure to live voice and encourages parents to produce words for their preterm infants (which can be difficult in the NICU). The benefits of book reading on language development have been largely investigated in full-term infants [69]. Despite less attention being devoted to exploring this topic with preterm infants, recently, Neri et al. [70] investigated possible advantages of book reading while in the NICU by comparing infants in a reading group with infants in a control group. Language and hearing skills showed an improvement in both groups. However, the reading group outperformed the control group. 

In sum, research demonstrates the importance of a nurturing environment to support language development in preterm infants. Although there is abundant literature investigating the causes underlying delayed language development in preterm infants compared with full-term infants, the literature is scant when considering the ways in which parents of preterm infants may support their language development at home, in everyday settings post-discharge from the NICU. Given that ID music and home musical activities have been shown to facilitate language acquisition in infants born at term [48,49,50], the present study will investigate the effects of musical enrichment on early language developmental outcomes. Given the importance of demographic and family variables in predicting language development, we will also test the effect of other enrichment interactions at home and individual differences in birth variables and parental stress on language acquisition.

### Aims and Hypotheses of the Current Study

With a focus on the earlier measurable phase of language acquisition and using parental reports, the aims of this study were as follows:

(i) Test the associations of crucial infant demographic information with language development, such as gestational age and weight at birth, as well as the corrected age (corrected age, or adjusted age, is a premature baby’s chronological age minus the number of weeks or months s/he was born early) at the time when completing the study. Gestational age and corrected age are expected to be positively correlated, and birth weight to be negatively correlated with language development.
(ii) Test the associations of parental variables with language development, such as perinatal stress [71] and parental education. Based on the literature reviewed above, parental education is expected to be positively correlated with infant language outcomes. The effects of perinatal stress are expected to be negatively (e.g., [72]) associated with the dependent measures.
(iii) Investigate the effects of two types of self-reported enrichment on language development, namely (i) home musical activities and, to control for the influence of non-musical enrichment and (ii) book readings and play stimulation. Language development was measured using the MacArthur–Bates Communicative Development Inventories: CDI-UK adaptation Words & Gestures form [73], suitable for the first phase of language acquisition. Specifically, the dependent variables derived from the the CDI were the Comprehension and Gestures scales (e.g., [74]). Based on previous research [48,49,50], we expect that home musical activities will be associated with language outcomes over and above the effect of other enrichment activities.

## 2. Materials and Methods

### 2.1. Design

This was a cross-sectional correlational study, where parents completed a survey that included relevant questionnaires for all variables of interest.

### 2.2. Participants

This study was conducted in London, UK. Native English-speaking participants of different nationalities (British, American, Caucasian, Australian, and Canadian) were recruited via social media such as Facebook and Instagram, as well as via the researchers’ personal and professional networks (e.g., <anonymised—Whittington Baby Charity, London>). Inclusion criteria were (i) participants had to be native English speakers/have English as the home language, (ii) their child was born <37-week gestation, and (iii) the child was, at the time of completing the survey, between 8 and 18 months corrected age. From the total number of individuals who accessed the survey, the following were excluded due to (i) having children outside the required age range (N = 10) and (ii) not completing the entire survey (N = 1223). A total of N = 145 were included in the analyses with a complete response set. A total of N = 117 comprised the main sample, without any suspected or identified areas of difficulties. A total of N = 28 comprised the sub-sample, with suspected or identified areas of difficulties such as sensory processing disorder, cerebral palsy, chronic lung disease, motor delay, blindness, speech delays, unsafe swallow, global development delay, brain injury, Dandy–Walker malformation, VACTERL association, hydrocephalus, and spina bifida. In order to avoid the introduction of potential confounding variables when considering language development, this sub-sample was analysed separately (Supplementary Materials). Therefore, the main analyses reported in Results included only the sample without areas of difficulty (N = 117; age mean = 13.15, range = 8.00–18.00, and SD = 3.43). Respondents were only female (100%), and they were the premature infants’ mothers—see information about the main sample in Table 1, Table 2 and Table 3.

### 2.3. Materials

After the demographic section, participants completed the following self-reports:

(i) The adaptation of the MacArthur–Bates CDI Questionnaire to British English [73], Words & Gestures form, suitable for infants aged 8–18 months. (Cronbach’s α = 0.99 for Comprehension and Production of Words and Cronbach’s α = 0.98 for Gesture). Example item: ‘Things children understand. In the list below, please mark the phrases that your child seems to understand. Are you hungry?’
(ii) Music@Home infant version [75], which is a psychometrically robust questionnaire for the assessment of the home musical environment, comprising 18 items in total, scored on a 7-point agreement scale (Cronbach’s α = 0.87). Items cover activities such as singing and playing with sound and instruments, including toys and parental beliefs. The questionnaire yields an overall Music@Home score (ranging from 18 to 126), with higher scores indicating higher levels of home musical activities. Example item: ‘I make music with my child (including toy instruments) almost every day’.
(iii) STIM-Q infant version [76] (Cronbach’s α = 0.89) was used to measure families’ general enrichment. Specifically, two scales were used that appear particularly relevant for language development: the ‘Reading’ scale (12 questions) referring to reading activities in the home environment, and ‘Parental Involvement in Developmental Advance’ (PIDA: 7 questions) measuring the number of different interactional activities occurring between caregiver and infant. Example item: ‘Do you have the opportunity to point to things around the house and name them for your child?’
(iv) The Perinatal PTSD Questionnaire (PPQ; Cronbach’s α = 0.86) [71] was used to investigate the level of stress and the presence of PTSD symptoms in caregivers. PPQ comprises 14 items scored on a five-point scale, ranging from 0 = not at all to 4 = often, more than a month (e.g., ‘Did you have bad dreams of giving birth or of your baby’s hospital stay?’). Higher scores are indicative of higher levels of stress/PTSD symptomatology. There are three subscales measuring intrusion, avoidance and hyperarousal symptoms.

### 2.4. Procedure

The questionnaires were inserted into a survey and distributed using the Qualtrics survey tool [77]. Caregivers were invited to complete the survey by clicking an online link, which was posted on social media and parent networks. Following the information section, participants were first required to give their informed consent and then complete the survey, which included demographic information. The completion of the survey took around 45–60 min, depending on individual differences in reading and decision time for responses and on the amount of detail that the respondents reported in the open question. However, the participants were informed that they had the opportunity to take short breaks during the completion of the survey, with their answers being saved. Respondents did not receive any compensation for completing the survey.

This research was approved by the Psychology Research Ethics Committee of <anonymise Middlesex University> (#15225) as conforming to the ethical principles of the British Psychological Society and the WMA Helsinki Declaration. All data were anonymised during collection, and respondents were informed that they could withdraw from the survey at any time by closing their browser.

### 2.5. Data Analysis

Data were analysed using SPSS version 23.0 [78]. Inspection of the UK-CDI Production scores revealed that most infants were mostly pre-verbal; thus, analyses investigating the relationship between musical home environment and CDI-UK focused on the CDI Comprehension and Gesture scales.

To test associations between infant variables (e.g., gestational age, birth weight, and days spent in the NICU), parental variables (parental stress, age, and education), and language development (CDI-Comprehension, Production, and Gesture), we performed correlational analyses.

To test the effect of informal musical interactions (as measured with the Music@Home) and other forms of enrichment, such as parental involvement in developmental advance and reading (as measured by the Stim-Q Infant), we constructed two multiple regression analysis models where each one of the language outcome variables was entered separately as the dependent variable (i.e., one model for CDI-Comprehension and one model for CDI-Gesture).

Since this research did not receive external funding and was conducted within a non-negotiable timeframe during the COVID-19 pandemic rather than a priori, a post hoc sensitivity power analysis was carried out before starting the statistical analyses. In order to determine if the sample of collected responses was large enough to detect reliable results, G*Power [79] was used to calculate a reliable medium effect size, with alpha ≤0.05 and power = 0.80 obtainable with a sample N = 117. Based on the smallest partial eta squared achieved for the Music@Home general factor effect in relation to the variable CDI-UK Comprehension in the present sample of infants without neurological complications (N = 117), the significant results in the CDI-UK Comprehension analysis showed power = 0.83, while the significant results in the CDI-UK Gesture analysis achieved power = 0.98. Thus, the sample for the present study can be considered sufficiently powered to detect reliable results [80].

## 3. Results

A summary of the scores from the questionnaires used and the relevant variables for the main sample (N = 117) is reported in Table 4.

The first interesting result is that infants scored within the normative profile for language development in their age group (between the 50th and 75th percentile) (Alcock et al., 2020 [73]): CDI-UK Comprehension mean = 162.18, range = 2.00–429.00; CDI-UK Gesture mean = 32.18, range = 2.00–75.00.

A second important result revealed that for 67.5% of the mothers, the mean PPQ score was high (M = 25.71) and well above 19, which is considered the cut-off for possible PTSD symptomatology.

Correlational analyses were conducted between all variables of interest: Music@Home general factor, the language development measures (CDI-UK Comprehension, CDI-UK Gesture, and CDI-UK Production), and STIMQ-reading, STIMQ-PIDA, PPQ-overall score, as well as Gestational age, birth weight, infants’ age (corrected), days spent in the NICU, maternal age, and education. The results are presented in Table 5.

Firstly, concerning the demographic variables, as expected, (i) the birth weight and gestational age at birth are negatively correlated with the days spent in the NICU, i.e., the higher the birth weight and gestational age, the fewer days infants spent in the NICU; (ii) both infants’ corrected age and maternal age are positively correlated with CDI-UK Comprehension scores, i.e., the older the infants and the mothers, the higher scores for CDI-UK Comprehension were reported to be, whereas only infant corrected age was associated with CDI Production; and (iii) gestational age, birth weight and infants’ corrected age were positively correlated with CDI-UK Gesture; the higher the infant’s gestational age, corrected age and weight, the better scores for CDI-UK Gesture were reported.

Concerning the effects of musical experience in the family, the Music@Home general factor was positively correlated with all three components of the CDI-UK measures, meaning that the more musical activities were performed in the home setting, the higher infants scored on CDI-UK Comprehension, Gesture, and Production scales. Additionally, maternal education was positively correlated with the Music@Home general factor, which means that the more educated the mothers were, the more musical activities they performed with their child at home. Moreover, the caregivers offered more general stimulation (STIMQ-PIDA) to the older infants, and this variable was also positively correlated with CDI-UK Comprehension and CDI-UK Gesture.

Lastly, maternal age is negatively correlated with the PPQ-overall score, suggesting that the younger mothers reported more symptoms of perinatal PTSD. Interestingly, no significant association emerged between perinatal stress (PPQ) and language measures, musical activities at home or general stimulation. However, a very weak association can be noticed, outlining a trend for a negative correlation between PPQ-overall score and CDI-UK Production and Gestures, with the more stressed mothers reporting lower scores for these two language development parameters.

Due to the lack of significant correlation between both STIMQ-reading and PPQ-overall score with the outcome variables, these variables were not entered into the subsequent analyses concerning language development. In preparation for further analyses, an independent group t-test was conducted to investigate whether Music@Home general factor scores (M = 98.77, SD = 15.10) differed when comparing younger and older infants using a median split for age to divide the sample into two similar groups. With Mdn = 14 months, there were N = 58 infants in the younger group (<14 months) and N = 59 in the older group (>14 months). The results were non-significant, *t* (115) = 0.993, *p* = >0.05, suggesting that overall, both groups were exposed to a similar level of musical activities in their home settings.

In order to develop a more general understanding of the effect of several variables found in the literature to be associated with language development in preterm infants, multiple regression analyses for predicting CDI-UK Comprehension and CDI-UK Gesture were performed for Music@Home general factor and STIMQ-PIDA including the following variables: infants’ age (corrected), gestational age, and birth weight. For each of the models, backward elimination was used, gradually eliminating variables with no significant contribution to the model. The final models reported are the most parsimonious and explanatory after progressively removing the different predictors. Before reporting, the data were checked and met the assumption of normality, linearity, homoscedasticity, and absence of multicollinearity.

The most parsimonious model for CDI-UK Comprehension (model 4) suggested that development was, predictably, associated with infant age (corrected), and specifically also with Music@Home general factor, independently from the other variables, which included infants’ demographic variables and STIMQ-PIDA (Table 6). A more nuanced pattern emerged for the development of CDI-UK Gesture (Table 7), for which, besides the above predictors, gestational age at birth also had a contribution (model 3).

## 4. Discussion

The present study was conducted online using parental reports and specifically investigated the influence of early musical experience, as measured by the Music@Home questionnaire [75], on language development measured by CDI-UK [73] while controlling for general enrichment at home (STIMQ) [76], as well as demographic variables.

The results suggested that, in spite of prematurity, infants’ language skills were not significantly delayed when compared to the normative scores reported in the country of data collection (UK) [73]. This result is in line with previous studies reporting non-significant differences between some groups of healthy preterm and full-term infants’ language abilities [81,82,83]. However, other literature has reported that, compared to full-term infants, preterm infants experience significant language delays [2,3,4,5,6,7]. Perhaps these inconsistent results could be explained by the fact that the studies which found significant delays have assessed a variety of preterm infants, including some with identified areas of difficulties, creating a confounding effect with prematurity on the outcomes. In contrast, the studies that did not find a significant difference in language skills between the two groups were mostly carried out with healthy preterm infants. The present study employed a similar practice, separating the group of infants with suspected or identified difficulties to avoid a confounding effect. Furthermore, an important factor in the present study is that the self-selected sample responding to the online survey was highly homogeneous, including highly educated mothers from professional environments. It is likely that this relatively privileged population had resources (both intellectual and financial) that supported their caregiving in compensating or overcoming the challenges associated with premature birth. Therefore, the finding that there is overall no delay in early language development for infants born preterm may not be replicated in a sample from less affluent environments. Nonetheless, this result can be regarded as extremely positive and encouraging, suggesting that providing caregivers of premature babies with information, and tools to facilitate and support communication and language development could significantly mitigate the disadvantage of prematurity.

Supporting our main hypothesis, the results showed that early language outcomes (comprehension and gestures) are predicted by the amount and variety of musical activities parents engaged their infants with in their home settings, even within this relatively homogeneous and highly educated middle-class sample. Interestingly, this effect was present across the age range studied here relatively and independently from both the expected increase in language abilities between 8 and 18 months and general enrichment in activities for infants. These results show, for the first time, that higher levels of home musical interactions facilitate early language outcomes in preterm infants, who are considered at risk of delays in language acquisition, thus identifying home musical activities as a protecting factor in this population, and suitable for recommending in intervention programmes. There is no reason to envisage that this prediction would not generalise to a less privileged social group. On the contrary, disadvantaged families suffering the challenges of premature birth might particularly benefit from support in an area of parenting that is part of the spontaneous parenting repertoire, with individual differences. By suggesting that an enriched home musical environment has direct implications for supporting word comprehension and gestural communication, the results are consistent with research that has been recently accumulating on how musical activities can enhance language development in infants [50,58] and also contribute to communication [47,84], as well as vocabulary, numeracy, attentional and emotional regulation in young children [85]

When considering the measured language outcomes separately, our analysis suggested that the development in CDI-UK Comprehension was predicted by the Music@Home general factor over and above the influence of age and independently from general enrichment in interactions with infants and other variables, which included infant demographics. Our analysis revealed a more nuanced pattern for CDI-UK Gesture, for which, besides the Music@Home general factor and the expected infant age (corrected) effect, gestational age at birth also contributed to predicting gesture scores, more explicitly, the lower the gestational age, the lower the gestural communication, but still not showing delays when comparing with norms. Studies have shown that preterm children acquire their early gestures at a slower pace than their full-term peers and that this difference is larger depending on prematurity [86,87]. Why gestural communication is particularly affected by gestational age is still relatively unclear, but findings indicate that this may be due to motor and general cognitive delays experienced by preterm infants [88]. It is also possible that gestures more readily reflect early communicative challenges, given that gestural communication gets established and consolidated before verbal utterances become predominant (see also Papadimitriou et al. [48] for similar trends in full-term infants).

In the present study, infants’ corrected age (rather than chronological age) at the time of completing the survey was used, as this is deemed to be the appropriate measure when assessing preterm infants’ development in order to accurately recognise genuine delays as opposed to perceived delays linked to infants gestational age at birth [89]. This is particularly the case in children up to 3 years old in order to avoid an underestimation of their abilities, considering preterm infants’ greater immaturity in their relation to the gestational age [90]. Our results were consistent with those reported in other studies also using corrected rather than chronological age, e.g., Fasolo et al. [91] and Suttora and Salerni [92] did not find a significant gap for gesture score when comparing their preterm and full-term samples. Conversely, there are studies in which, even when infants’ corrected age is considered, they still experience language delays from a phonological point of view [15] as well as in comprehension [93]. Furthermore, a variety of studies have used both chronological and corrected age. For example, Cattani et al. [94] investigated the language development of preterm children longitudinally, from 12 to 24 months, using both chronological and corrected age using the MacArthur–Bates CDI, Italian adaptation [95] as a measure. Their results revealed no delays for gesture scores when corrected age was considered, but when chronological age was considered, the scores fell between the 27th and 33rd centile. This suggests that for a more nuanced picture, future studies may adopt a developmental trajectories approach [96,97,98] in which both chronological and corrected age are considered longitudinally.

When considering perinatal stress, it was found that the younger the mothers, the more stressed they reported to be (consistent with Chifa et al.) [24]. The transition to motherhood is a major, challenging development in a woman’s life, followed by many changes that require resources and continuous learning. However, the literature is mixed in regards to the link between maternal age and levels of stress. Some studies suggest that older mothers experience more depressive symptoms [99] while others suggest that the transition to motherhood for young mothers might be linked with many additional challenges compared to older new mothers [100], such as the need to manage multiple life changes at the same time, the shift to adulthood, a possible marriage, career choices, as well as motherhood [101]. These can heavily add to feeling overwhelmed by the responsibilities that having a child carries (especially the first child), such as caring for the infant, dealing with issues concerning safety, colic, or choking, as well as trying to keep the marital relationship working [102]. But besides these general aspects, being a mother of a preterm infant requires functioning under high levels of stress and pressure: young mothers possibly have not yet developed such abilities, hence being negatively affected more or for longer. For example, mothers of preterm infants experience uncertainty, helplessness, role alternation, and possibly, being an outsider, hence isolation [103].

Contrary to our initial hypothesis, perinatal stress and PTSD symptoms reported by the mothers were not significantly correlated with CDI-UK aspects, in spite of the caregivers showing very high levels of stress (on average, above the cut-off for clinically relevant scores). This means that despite being very stressed, mothers did what was necessary to best support their infants’ language acquisition. Their personal difficulties did not impact in a negative way on their infant’s communication skills, contrary to the body of work suggesting that mothers’ mental health is negatively associated with infants’ language skills [104]. Perhaps the lack of a statistically significant relationship between maternal PPQ score and infant UK-CDI might be partially explained by the unexpectedly homogeneous sample, representative of the upper-middle class, with socioeconomic stability and very high educational levels. Thus, these results are consistent with previous studies showing a positive relationship between SES and language development in preterm children [105,106]. Nevertheless, a very weak trend was identified: the more stressed mothers were, the lower scores were reported for CDI-UK Comprehension and CDI-UK Production. This non-significant trend needs to be followed up in future research due to the limitation of having mostly highly educated, middle-class respondents in the present study. It is likely that higher educational attainments enable mothers to understand the importance of information and possibly seek out further resources and experiences on how to best support their infants’ language development and respond to their special needs. Indeed, studies have suggested that parental responsivity is further linked with better language outcomes in children [12,107]. This would suggest that caregivers of preterm infants from disadvantaged environments might benefit from specific parenting workshops dedicated to prematurity.

In terms of the benefits of music, it is essential to note that parents play a crucial role in nurturing their children’s musical development [108], beginning in early infancy with parents singing to and with a child [109]. Considering the high amount of musical engagement reported by the mothers in the present study, it is impossible not to think of the potential benefits that musical activities have had for the parents, too. For example, music strengthens parent–child relationships through interaction, consolidates bonding, helps to extend the repertoire of parenting skills, offers a feeling of reward for helping their children to meet developmental milestones [110], and ameliorates anxiety and stress [111]. Similarly, in Chifa et al. [24], parents reported that music induced a state of relaxation and comfort for both their preterm infants and them while in the NICU, helped to bond with the baby, positively impacted infants’ development and had a key role in the adaptation to the home environment after discharge from the NICU. This is potentially another aspect that helped parents deal with their mental health without letting it impact infants’ language. Therefore, this is an interesting area to further explore in future research, which may also elucidate the indirect benefits of musical interactions for infants (i.e., via benefits for the caregivers). Indeed, in the context of premature birth, various studies have also investigated the benefits of music therapy [112] and music exposure [113] from the NICU to post-discharge. There is substantial evidence showing that early music-based interventions have benefits on premature infants’ development, such as feeding, sleeping, weight gain, and improvement of vital signs, [114,115]. Music intervention is effective in stimulating early language development, as well as inducing functional links between the auditory cortex and other brain areas connected with music processing [116], and is beneficial for infant–parent attachment [117], which is also important for language development [118]. Our study significantly adds to these findings by providing the first evidence that informal and unstructured musical engagement in families of premature infants may impact their language development.

A few limitations must be considered. Firstly, the sample in our study was unexpectedly homogeneous, affluent and highly educated, representative of the upper-middle class, with socioeconomic stability, which does not offer the opportunity to further extend the interpretability of the results to the general population of preterm infants’ caregivers, including low SES and low educational attainments. Secondly, our sample was also limited by gender bias–only mothers took part in the survey. Fathers have not been represented despite a substantial increase in fathers’ involvement in childcare or even becoming the main caregiver in recent years [119]. Thirdly, we have not collected information on where the participants resided, and it is possible that some of our participants resided outside of the UK. However, irrespective of country of residence or nationality, the infants would have been exposed mainly to the English language in their home environment. Lastly, we did not collect information regarding respondents’ inclination towards music and/or musical experience and acknowledge that participants with a propensity towards music would have been more likely to complete the survey. In spite of the acknowledged limitations, the present study provides important insights into the activities that preterm infants can engage with within their home settings to support their language development.

## 5. Conclusions

In sum, the present study has revealed novel findings in a crucial area of early development (language) for a population with known delays in this area: prematurely born infants. The study highlights a significant positive association between language development (comprehension and gesture) and home-based musical interactions. This finding has the potential to inspire the use of music in the early years and generate considerable societal impact, music being a non-pharmacological, low-cost intervention that any family, regardless of their social status, can include in their daily routines.

There are a number of future directions stemming from the present study. For instance:

(i) This study used a simultaneous design, i.e., measures of home musicality and language development were taken at the same point in time. However, it would be important that further research adopts a longitudinal design [120], which could help with identifying crucial time windows for intervention. For example, caregivers of pre-term infants could provide data on home musical and other activities at 6, 14, and 20 months of age of their children, and the relationship of these measures could be studied in association with their early developing language. Indeed, it has been suggested that parental singing at 6 months predicts early language outcomes at 14 months in full-term infants [46]. Initial work could be conducted using online surveys (quantitative) and parental diaries (qualitative), but remote observational methods could be also employed.
(ii) The participants in this study were predominantly highly educated and representative of the middle social class. Therefore, it would be interesting to conduct a similar study, recruiting mainly participants from lower social classes, in order to gain a deeper understanding of the impact that social class has on language development.
(iii) An important implication of the present study is the need to create a partnership with relevant institutions, such as NICUs, baby groups and early childcare settings. This partnership could aim to develop workshops, dedicated to caregivers of preterm infants for developing musicality at home, as well as to provide parents with the information required to facilitate access to those activities (e.g., baby music groups).

## Figures and Tables

**Table 1:** General sample characteristics (parents and infants) (N = 117).

	M	SD	Min	Max
Maternal age (years)	31.03	5.65	20	44
Gestational age at birth of the children (weeks)	30.60	3.54	22	36
Days spent in the NICU (N)	47.71	49.89	0	242
Infant birth weight (g)	1542.85	639.60	368	3040
Corrected age of the infants when the survey was completed (months)	13.38	3.39	8	18

**Table 2:** Infants’ sample characteristics (N = 117).

Gender of the Children	Siblings	Categories of Prematurity	Categories of Birth Weight	Status
55 (47.0%) Female	43 (36.8%) Yes	31 (26.5%) Extremely preterm	31 (26.5%) ELBW	95 (81.2%) Singleton
62 (53.0) Male	74 (63.2%) No	39 (33.3) Very preterm	27 (23.1) Very low birth weight	21 (17.9%) Twins
		47 (40.2) Moderate to late preterm	53 (45.3) Low birth weight	1 (0.9%) Triplets
			6 (5.1) Normal birth weight	

Note: The categories of prematurity are defined as follows: extremely preterm–infant born below 28-week gestation; very preterm–infant born between 28 and 32-week gestation; moderate to late preterm–infant born between 32 and 37-week gestation. The categories of birth weight are defined as follows: extremely low birth weight (ELBW)–less than 1000 g; very low birth weight–less than 1500 g; low birth weight–less than 2500 g; normal birth weight–over 2500 g.

**Table 3:** Parents’ sample characteristics (N = 117).

Gender of the Respondents	Nationality of Participants	Highest Level of Education Achieved	Years Spent in Education	Occupational Groups of Respondents
117 (100%) Female	81 (69.2 %) British	3 (2.6%) Post-graduate (PhD or doctorate)	50 (42.7%) 14–18	34 (29.1%) Intermediate managerial/professional
	18 (15.4%) American	28 (23.9%) Post-graduate (Master’s degree or equivalent)	29 (24.8%) Over 18	31 (26.5%) Supervisory or clerical/junior managerial
	7 (6%) Caucasian	61 (52.1%) College or University	17 (14.5%) 12–14	18 (15.4%) Skilled manual worker
	5 (4.3%) Australian	17 (14.5%) Higher or secondary education	16 (13.7%) 9–12	10 (8.5%) Higher managerial/administrative
	6 (5.1%) Canadian	8 (6.9%) Secondary school up to 16 years of age	4 (3.4%) 6–9	9 (7.7%) Homemaker
			1 (0.9%) Less than 6	15 (12.8%) Other

**Table 4:** Descriptive statistics for all the relevant variables (N = 117).

	Mean	Median	Std. Dev	Range
CDI-UK Comprehension	162.18	129.00	123.11	2.00–429.00
CDI-UK Gesture	32.18	32.00	19.03	2.00–75.00
CDI-UK Production	39.35	10.50	72.99	0–397.00
Music@Home general factor	98.77	99.00	15.10	66.00–126.00
STIMQ: reading scores	12.31	12.00	2.81	3.00–19.00
STIMQ: PIDA scores	5.52	6.00	1.41	1.00–7.00
PPQ: overall scores	25.71	28.00	12.18	0–50.00
PPQ: intrusiveness	5.68	5.00	3.29	0–12.00
PPQ: avoidance	9.06	9.00	4.86	0–19.00
PPQ: arousal	10.96	11.00	5.77	0–20.00
Gestational age (weeks)	30.60	31.00	3.54	22.00–36.00
Birth weight (grams)	1542.85	1559.00	639.60	368.00–3040.00
Days spent in the NICU	47.71	30.00	49.89	0–242.00
Maternal education (levels ^1^)	4.69	5.00	1.14	1.00–6.00
Corrected infants’ age (months)	13.15	14.00	3.43	8.00–18.00

Note: CDI—Communicative Development Inventory; M@H—Music@Home. STIMQ—Cognitive Home Environment Questionnaire. PIDA—Parental Involvement in Developmental Advance. PPQ—Perinatal PTSD Questionnaire. ^1^ This was an ordinal variable where 1 = 6–9 years in education, 2 = 9–12 years, 3 = 12–14 years, 4 = 14–18 years, and 5 = over 18 years.

**Table 5:** Bivariate correlation between relevant variables (N = 117).

	1	2	3	4	5	6	7	8	9	10	11	12	13
1. Gestational age													
2. Birth weight	0.85 **												
3. Infants’ age (corrected)	0.13	0.08											
4. Days spent in the NICU	−0.81 **	−0.67 **	−0.06										
5. Maternal age	0.07	0.07	−0.12	−0.04									
6. CDI-UK Comprehension	0.09	0.05	0.61 **	−0.06	−0.28 **								
7. CDI-UK Production	0.11	0.08	0.51 **	−0.09	−0.18	0.66 **							
8. CDI-UK Gesture	0.26 **	0.21 *	0.78 **	−0.14	−0.11	0.82 **	62 **						
9. M@H general factor	−0.01	0.05	0.17	0.08	0.04	0.25 **	0.20 *	0.33 **					
10. STIMQ-reading	0.05	−0.01	0.13	0.04	−0.02	0.08	−0.07	0.17	0.00				
11. STIMQ-PIDA	0.12	0.08	0.39 **	−0.11	−0.07	0.27 **	0.17	0.38 **	0.21 *	0.38 **			
12. PPQ-overall score	−0.01	0.03	−0.05	0.04	−0.32 **	0.01	−0.11	−0.12	0.00	0.11	0.16		
13. Maternal education	0.11	0.09	−0.07	−0.07	0.35 **	0.08	0.05	0.13	0.21 *	0.04	−0.07	−0.06	

* *p* < 0.05; ** *p* < 0.01. Note: CDI—Communicative Development Inventory. M@H—Music@Home. STIMQ—Cognitive Home Environment Questionnaire. PIDA—Parental Involvement in Developmental Advance. PPQ—Perinatal PTSD Questionnaire.

**Table 6:** Multiple regression results for CDI-UK Comprehension, Music@Home general factor, infants’ age (corrected), gestational age, birth weight, and STIMQ-PIDA.

	*β*	*t*	*p*	*R* ^2^	*F*	*p*
Model 1				0.39	14.28	0.000
Music@Home general factor	0.15	1.99	0.04			
Infants’ age (corrected)	0.57	7.53	0.00			
Gestational age	0.07	0.49	0.62			
Birth weight STIMQ-PIDA	−0.06 −0.01	−0.45 −0.20	0.65 0.84			
Model 4				0.39	36.41	0.000
Music@Home general factor	0.15	2.06	0.04			
Infants’ age (corrected)	0.58	7.80	0.00			

**Table 7:** Multiple regression results for CDI-UK Gesture, Music@Home general factor, infants’ age (corrected), gestational age, birth weight, and STIMQ-PIDA.

	*β*	*t*	*p*	*R* ^2^	*F*	*p*
Model 1				0.68	48.26	0.000
Music@Home general factor	0.21	3.87	0.00			
Infants’ age (corrected)	0.72	13.15	0.00			
Gestational age	0.18	1.81	0.07			
Birth weight STIMQ-PIDA	−0.02 0.02	−0.19 0.40	0.84 0.68			
Model 3				0.68	81.67	0.000
Music@Home general factor	0.21	3.91	0.00			
Infants’ age (corrected)	0.72	13.45	0.00			
Gestational age	0.16	3.13	0.00			

## Data Availability

Data are available from the corresponding author upon reasonable request. The data are not publicly available due to the participants not being informed about public data deposit procedures at the time of completing the study.

## Supplementary Materials

The following supporting information can be downloaded at: https://www.mdpi.com/article/10.3390/children11050542/s1.
