# Supplementary material for: Home Musical Activities Boost Premature Infants’ Language Development
Source: Children (Basel). 2024 May 1;11(5):542. doi: 10.3390/children11050542 (PMC11120229; doi:10.3390/children11050542)
Supplement: Supplementary file 1 [file children-11-00542-s001.zip › children-2775068-supplementary.pdf]

Analysis of the sub-sample of infants with suspected or identified impairments.

A group of (n=28, age mean=14.54, range=8.00-18.00 and SD=3.52) participants were analysed separately due to the infants having suspected or identified impairments, such as sensory processing disorder, cerebral palsy, chronic lung disease, motor delay, blindness, speech delays, unsafe swallow, global development delay, brain injury, Dandy Walker malformation, VACTERL Association, Hydrocephalus, spina bifida (all details in Table 2). Since these impairments may be associated with further delay or disorder in the development of language, this sample was analysed separately to avoid introducing potential confoundings in the main analyses. Although this sample is small, it was deemed important to explore whether some of the trends emerged in the main sample were also found in this disadvantaged sub-sample of premature infants.

As can be seen in Table SM1, the scores for CDI-UK are predictably slightly lower in comparison to the main sample (reported in Table 4). The mothers' PPQ-overall scores are very similar to those of the main sample, thus reporting very high levels of perinatal stress/trauma. Additionally, Music@Home and STIMQ scores are similar to the main sample, suggesting similar patterns of engagement with musical activities and other cognitive stimulating activities across this sub-sample and the main sample.

Table S1. *Descriptive statistics for the predictors and outcome variables for the sub-sample (N=28) of premature infants with suspected or identified impairment*

	**Mean**	**Median**	**Std. Dev**	**Range**
CDI-UK Comprehension	142.03	93.50	137.57	2.00-424.00
CDI-UK Gesture communication	29.14	25.00	19.51	0-70.00
CDI-UK Production	31.78	9.00	79.69	1.00-380.00
Music@Home: General Factor	93.57	92.00	15.56	66.00-123.00
Music@Home: Parental Initiation of Singing	25.92	26.00	5.32	11.00-35.00
STIMQ: Reading scores	11.57	11.50	3.36	5.00-18.00
STIMQ: PIDA scores	5.00	5.00	1.86	0-7.00
PPQ: Overall scores	24.82	27.00	11.75	3.00-49.00
PPQ: Intrusiveness	5.60	5.50	3.29	0-12.00
PPQ: Avoidance	9.07	9.00	4.64	0-19.00
PPQ: Arousal	10.14	10.00	4.86	0-20.00
Gestational age (weeks)	30.54	31.50	4.29	2.00-37.00
Birth weight (grams)	1481.00	1365.00	749.49	453.00-3120.00
Infants` age (months)	14.54	16.00	3.52	8.00-18.00
Maternal age (years)	31.61	31.00	5.76	21.00-44.00

Bivariate pilot correlations were tentatively conducted as exploratory analyses for future studies, and the results are presented in Table SM2.

Table S2. *Bivariate correlations between language development, Music@Home, STIM-Q and relevant demographic variables for the sub-sample (N=28) with infants with suspected or identified impairment*

		1	2	3	4	5	6	7	8	9	10	11
**1**	Gestational age											
**2**	Birth weight	.86**										
**3**	Infants` age (corrected)	.16	.11									
**4**	Maternal age	-.17	-.10	-.49**								
**5**	CDI-UK Comprehension	.46*	.36	.28	.13							
**6**	CDI-UK Productive	.29	.40	-.36	.49*	.52*						
**7**	CDI-UK Gesture	.44*	.37	.35	-.09	.89**	.51*					
**8**	M@H-General Factor	-.06	-.07	-.02	.40*	.47*	.21	.37				
**9**	STIMQ-Reading	-.09	-.12	.02	.12	.09	-.33	-.06	.13			
**10**	STIMQ-PIDA	-.14	-.11	.30	-.10	.13	-.57**	.14	.12	.43*		
**11**	PPQ-Overall Score	.05	.07	.01	-.06	.14	-.34	.15	.23	.30	.56**	

* $p < 0.05$; ** $p < 0.01$.

Concerning the demographics, as expected, gestational age was positively correlated with birthweight and with CDI-UK, with better scores in both Comprehension and Gesture associated with later gestational age. Maternal age was positively correlated with CDI-UK Productive and Music@Home: General Factor, suggesting that the older the mothers, the more home musical activities they engage their infants with and the higher the scores for CDI-UK Production they report.Critically for this study, Music@Home: General Factor was significantly positively associated with CDI-UK Comprehension, even in this disadvantaged group, which outlines the importance of such activities for language development in early years.

Interestingly, PPQ-overall score was positively correlated with the STIMQ-PIDA, which means that the more stressed the mothers were, the more activities for infants' development they performed, possibly suggesting overstimulation. Consistently, a negative trend can be observed between PPQ-overall score and CDI-UK Production, with the higher the stress levels reported, the lower infant productive vocabulary observed. which might suggest that maternal stress could negatively impact infants` production language in this disadvantaged sample. However, unlike the findings in the main sample, STIMQ-PIDA was positively correlated with CDI-UK Production, suggesting that the more developmental activities parents engaged their children with, the more productive vocabulary was reported.

In the sub-sample of participants with suspected or confirmed areas of difficulties, language scores were generally lower, possibly due to various impairment, and not only to prematurity. With the relatively low number of participants (n=28), unfortunately, it was not possible to carry out an in-depth exploration of the data. However, interestingly, and in spite of the exploratory nature of the analyses conducted, it was found that the more musical activities infants were exposed to in their home settings, the higher scores their caregivers reported for CDI-UK Comprehension, similarly to the main sample. Although these can only be considered pilot results, they are very encouraging in suggesting that language development gains may occur in this disadvantaged sample when caregivers provide an enhanced musical home experience. This means that recommendations can be drawn from the present study to support infants and families in this impaired group. This may include the implementation of parenting musical skills workshops whereby the importance of musical activities for development would be explained to the parents along with examples of good practice.
